# A Negative Index Nonagonal CSRR Metamaterial-Based Compact Flexible Planar Monopole Antenna for Ultrawideband Applications Using Viscose-Wool Felt

**DOI:** 10.3390/polym13162819

**Published:** 2021-08-22

**Authors:** Kabir Hossain, Thennarasan Sabapathy, Muzammil Jusoh, Mahmoud A. Abdelghany, Ping Jack Soh, Mohamed Nasrun Osman, Mohd Najib Mohd Yasin, Hasliza A. Rahim, Samir Salem Al-Bawri

**Affiliations:** 1 Advanced Communication Engineering (ACE), Centre of Excellence, Universiti Malaysia Perlis (UniMAP), Jalan Tiga, Pengkalan Jaya Business Centre, Kangar 01000, Malaysia; hossain.kabir42@gmail.com (K.H.); muzammil@unimap.edu.my (M.J.); pingjack.soh@oulu.fi (P.J.S.); nasrun@unimap.edu.my (M.N.O.); najibyasin@unimap.edu.my (M.N.M.Y.); haslizarahim@unimap.edu.my (H.A.R.); 2 Faculty of Electronic Engineering Technology, Kampus Alam UniMAP Pauh Putra, Universiti Malaysia Perlis (UniMAP), Arau 02600, Malaysia; 3 Electrical Engineering Department, College of Engineering, Prince Sattam Bin Abdulaziz University, Wadi Addwasir 11991, Saudi Arabia; 4 Department of Electrical Engineering, Faculty of Engineering, Minia University, Minia 61519, Egypt; 5 Centre for Wireless Communications (CWC), University of Oulu, P.O. Box 4500, 90014 Oulu, Finland; 6 Space Science Centre, Climate Change Institute, Universiti Kebangsaan Malaysia, Bangi 43600, Malaysia; s.albawri@gmail.com

**Keywords:** metamaterials, high-performance textiles, wearable antenna, textile antennas, polymer

## Abstract

In this paper, a compact textile ultrawideband (UWB) planar monopole antenna loaded with a metamaterial unit cell array (MTMUCA) structure with epsilon-negative (ENG) and near-zero refractive index (NZRI) properties is proposed. The proposed MTMUCA was constructed based on a combination of a rectangular- and a nonagonal-shaped unit cell. The size of the antenna was 0.825 λ_0_ × 0.75 λ_0_ × 0.075 λ_0_, whereas each MTMUCA was sized at 0.312 λ_0_ × 0.312 λ_0_, with respect to a free space wavelength of 7.5 GHz. The antenna was fabricated using viscose-wool felt due to its strong metal–polymer adhesion. A naturally available polymer, wool, and a human-made polymer, viscose, that was derived from regenerated cellulose fiber were used in the manufacturing of the adopted viscose-wool felt. The MTMUCA exhibits the characteristics of ENG, with a bandwidth (BW) of 11.68 GHz and an NZRI BW of 8.5 GHz. The MTMUCA was incorporated on the planar monopole to behave as a shunt *LC* resonator, and its working principles were described using an equivalent circuit. The results indicate a 10 dB impedance fractional bandwidth of 142% (from 2.55 to 15 GHz) in simulations, and 138.84% (from 2.63 to 14.57 GHz) in measurements obtained by the textile UWB antenna. A peak realized gain of 4.84 dBi and 4.4 dBi was achieved in simulations and measurements, respectively. A satisfactory agreement between simulations and experiments was achieved, indicating the potential of the proposed negative index metamaterial-based antenna for microwave applications.

## 1. Introduction

Flexible substrates including organic substances, such as polymers, paper, plastics, textiles, and fabrics, have become increasingly important to enable increased flexibility in wearable sensors/antennas [1]. Flexible antennas consist of a dielectric material (which works as the substrate) and a conductive material (which can be used as a radiating element and/or ground plane). Pure metals, metals mixed with fabrics, and conductive inks [2] are examples of materials that can be used as conductive materials. Meanwhile, polymers such as foam, paper textile fabrics, plastics, and soft printed circuit boards (PCBs) are all common dielectric polymer materials. Other than dielectric polymers, extensive research has been conducted on conductive polymers. They are explored for various applications such as solar energy harvesting [3], tissue engineering [4], supercapacitor design [5,6,7], gas sensors [8], and immunosensors [9]. In particular, flexible conductive polymers were also proposed by [10,11]. However, in antenna designs, more concerns are directed to the dielectric of the antenna since the antenna performance is mainly determined by the electrical characteristic and the mechanical flexibility of the dielectric substrate. Recent developments in manufacturing techniques for flexible polymer antennas are appealing due to the low permittivity with low losses [12]. In flexible antenna designs, dielectric polymer materials which are commonly used as substrates are classified either as natural polymers (e.g., rubber, silk, wool) or synthetic/human-made polymers (e.g., polystyrene, polyvinylchloride, nylon) [1]. Textile structural composites (which are considered as natural materials) are versatile in terms of their exceptional physical and mechanical properties which can be adopted in particular engineering applications to meet the desired requirements [13]. 

Ultrawideband (UWB) technology has triggered enormous research attention in wireless communications, especially after the allocation of the unlicensed frequency band (3.1 to 10.6 GHz) by the Federal Communications Commission (FCC) in 2002 [14]. A UWB antenna has the capability of providing high-speed data transmission with low-power spectral densities compared to conventional wireless communication systems within short distances. The application of UWB has been expanded into the wireless body area network (WBAN) domain based on the IEEE 802.15.6 WBAN standard [15,16]. Recent technological developments have resulted in compact and smart biomedical sensors/antennas for implementation on the human body. These antennas and sensors are most ideal for implementation in WBAN-type networks, as they are useful in sectors such as wearable computing, health monitoring, rescue systems, and patient tracking [17,18]. These applications require wireless devices to be placed close to the human body, which demands antennas and sensors to be developed using flexible materials. To prolong their usage near or on the body and, at the same time, ensure the safety and comfort of the user, they are best to be integrated onto clothing. Recently, conductive textiles have been introduced commercially, spurring the design of antennas for WBAN using textiles [16].

Sensors and smart devices have been the subject of extensive research over the past decade, with the goal of making them more easily integrated onto the human body [19]. Fabrics have been used as a natural and comfortable substrate for wearable electronic devices. Fabrics can now contain electrical functionality due to miniaturization of electronic components and innovative technologies [20]. There has been a lot of recent research on cloth fabrics, including sewn textiles, embroidered textiles, nonwoven textiles, knitted fabrics, woven fabrics, printed fabrics, braiding, laminated fabrics, spinning, and chemically treated fabrics [21]. Developing modern textile-based sensors has become a substantial undertaking in recent years, with numerous studies focusing on applications such as athletic training [22], emergency rescue and law enforcement [23], fitness monitoring [24], and other fields. 

Metamaterials (MTMs) are artificial composite structures with exotic electromagnetic properties which can be used for potential groundbreaking applications (e.g., in antenna design, subwavelength imaging) [25]. Consequently, MTMs are suitable to be applied to improve WBAN antennas in terms of gain, radiation patterns, bandwidth (BW), and size compactness [26,27,28]. The characteristics of MTMs can be single negative (SNG) or double negative (DNG) based on the dielectric permittivity (*ε*) or magnetic permeability (*μ*). For SNG MTMs, either *ε* or *μ* can be negative, and for DNG MTMs, both *ε* and *μ* are negative. For SNG MTMs, if *ε* is negative, they are called epsilon-negative (ENG) MTMs, and if *μ* is negative, they are called mu-negative (MNG) MTMs [17,29]. Furthermore, the refractive index of a material depends on the *ε* and *μ*, which defines the extent of reflection and refraction [30]. However, the near-zero refractive index (NZRI) property can enhance the gain, as reported in [31]. Several metamaterial structures have been proposed in terms of complementary split-ring resonators (CSRR) [26,32], split-ring resonators (SRRs) [33], planar patterns, and capacitance-loaded strips (CLSs) [29]. Some other MTM structures such as electromagnetic bandgaps (EBGs) and artificial magnetic conductors (AMCs) were discussed in [27]. Such metamaterial-based UWB antennas have been reported in the literature with proven antenna performance enhancements [27,28]. In [34], MTMs were loaded into UWB wearable antennas for non-invasive skin cancer detection. Likewise, in [35], the proposed MTM UWB antenna was used for breast cancer detection. Despite the different designs, antennas for wearable applications should be compact, low cost, lightweight, and able to be integrated into circuits with ease [28]. When constructing metamaterials for metamaterial-enhanced devices, it is crucial to take into consideration the fabrication difficulty. Therefore, when developing textile-based metamaterials, extra care should be taken in each design phase [21,36].

This paper proposes a compact textile antenna incorporated with an MTM unit cell array (MTMUCA) structure, with an in-depth analysis. A polymer-based viscose-wool felt was adopted as the dielectric material of the antenna. The felt is a composite material that is developed from a naturally available polymer, wool, and a human-made polymer, viscose, that is derived from regenerated cellulose fiber. An equivalent circuit model was developed to present the working principles of the overall structure. Its structure was simulated and validated experimentally from 1 to 15 GHz. First, the transmission–reflection (RTR) method was used to extract the effective parameters of the MTMs in this work. The simulation results indicate that the MTM unit cell (MTMUC) and the MTMUCA are almost identical in performance, with an ENG BW of at least 11.53 GHz and an NZRI BW of 8.5 GHz. To the best of the authors’ knowledge, the design of such textile-based ENG/ NZRI incorporated with a flexible MTM array antenna is yet to be reported in the literature. A comparison of the proposed antenna with similar designs in the literature is presented in Table 1. 

## 2. Flexible Polymer-Based Textile Antenna Design with Metamaterial

In this work, the proposed MTM antenna was simulated and fabricated on textile materials. Shieldit Super^TM^ with a thickness of 0.17 mm and a conductivity value of 1.18 × 10^5^ S/m was used as the ground plane and the radiator. Meanwhile, a 3 mm viscose-wool felt substrate with a dielectric constant of 1.44 and loss tangent of 0.044 was employed. The choice of the viscose-wool felt was mainly due its strong Shieldit Super^TM^–polymer adhesion. This type of flexible polymer is also easily available on the market; thus, no special treatment was required in developing the material in the lab, as required by other polymers such as polydimethylsiloxane (PDMS) [2]. The felt contained 30% viscose and 70% wool that formed a good composition of fibers with a density of 0.25 gm/CC. This property can help the Shieldit Super^TM^ easily iron out and attach to the polymer felt [36,41]. Computer Simulation Technology’s (CST) Microwave Studio Suite (MWS) was used to model and simulate the MTM and MTM-integrated antenna over the frequencies of interest from 1 to 15 GHz. The analyses of these structures are reported in the following subsections.

### 2.1. Metamaterial Design

**Figure 1:**
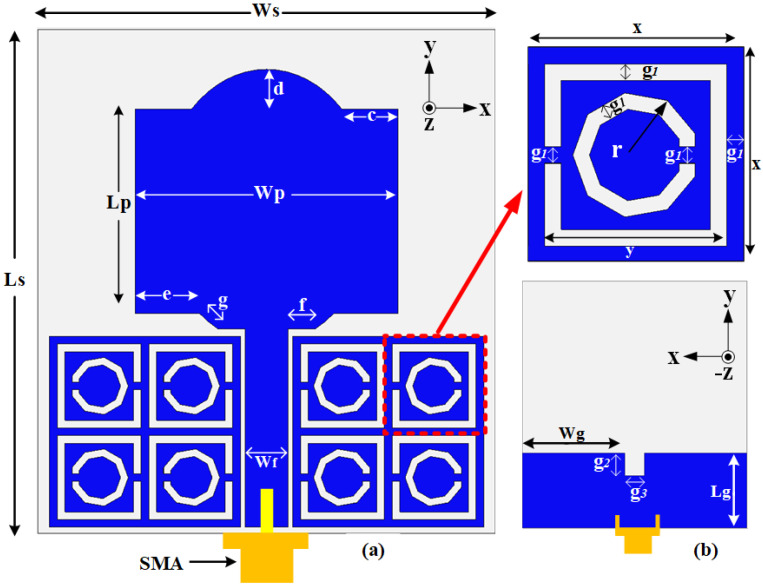
Schematic diagram of the proposed antenna and the MTMUCA structure (flexible polymer in gray and flexible conductive element in blue): (**a**) front view; (**b**) rear view.

**Figure 2:**
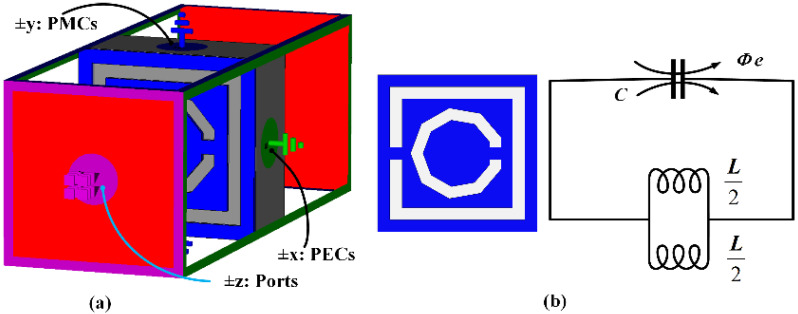
(**a**) 3D view of the MTMUCA simulation setup. (**b**) Topology of the MTMUC structure and its equivalent circuit model, where C = capacitance of MTMUCA, L/2 = each inductance (blue indicates the metallized areas).

The proposed MTMUCA was designed based on a CSRR structure and is illustrated in Figure 1a. Its overall size was 12.5 × 12.5 × 3 mm^3^, and other dimensions are summarized in Table 2. A square loop and a nonagonal-shaped structure were combined to form the MTMUC structure. To characterize the metamaterial unit cell, the MTMUC structure was placed between two waveguide ports on the positive and negative *z*-axis and was excited with a transverse electromagnetic (TEM) wave, as depicted in Figure 2a. It was bounded by a perfect electric conductor (PEC) boundary at the *±x*-axis and a perfect magnetic conductor (PMC) boundary at the *±y*-axis. A frequency solver with a tetrahedral mesh scheme was utilized in simulations over the frequency of interest. In this metamaterial simulation, the unit cell was without a conductive layer at the bottom [42].

The MTMUC structure and its equivalent circuit model are depicted in Figure 2b, where ohmic losses are unaccounted for [43]. The MTM modeled on a transverse plane acts as an *LC* resonator, which can be excited by the orthogonal electric field. Conversely, this structure behaves similarly to an electric dipole when excited by an axial electric field. The primary resonance can also be excited by the external magnetic field along the *y*-axis, as the CSRR can also exhibit a magnetic behavior [43,44]. The SNG properties can be tailored by appropriately modeling the CSRR gaps into the design. The surface current distribution of the proposed MTM was extracted for further study based on the setup exhibited in Figure 2a.

**Figure 3:**
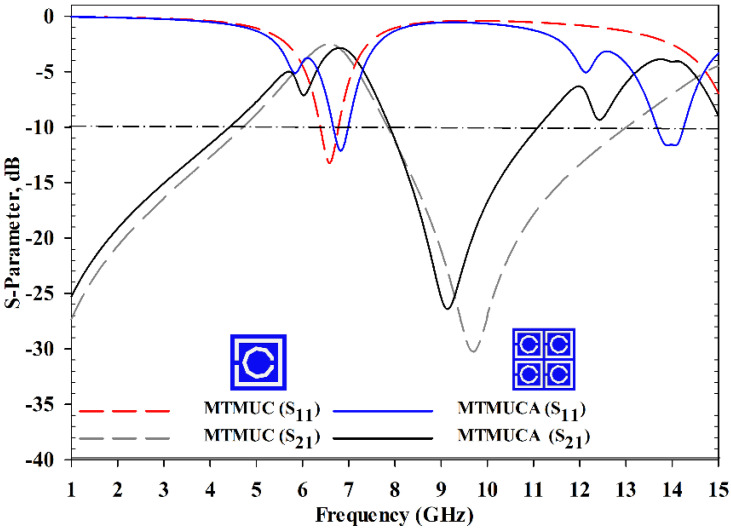
S-parameter of the MTMUC and MTMUCA structures.

The simulated S-parameter of the MTMs is shown in Figure 3. In prior analyses, the material effective parameters (e.g., permittivity, refractive index) and MTM’s stopband behavior were investigated. The transmission coefficient (*S*_21_) of the MTMUC structure ranged from 1 to 4.65 GHz, and from 7.53 to 11.71 GHz, and the reflection coefficient (*S*_11_) ranged from 6.34 to 6.68 GHz, and from 13.83 to 14.44 GHz. On the other hand, the MTMUCA showed an operational *S*_21_ from 1 to 4.45 GHz, and from 7.84 to 11.18 GHz, whereas the *S*_11_ was from 6.34 to 7.01 GHz, and from 13.65 to 14.26 GHz. Hence, the stopband behaviors clearly satisfied the *S*_21_ ≤−10 dB requirement. 

The RTR [28,45] method was employed to extract the effective parameters from the normal incident scattering parameters using (1) to (7), starting with calculating *S*_11_ and *S*_21_ from Equations (1) and (2) as follows:

(1) $$S_{11}=(\frac{R_{01}(1-e^{i2nk_{0}d})}{1-R_{01}{}^{2}e^{i2nk_{0}d}})$$

(2) $$S_{21}=(\frac{(1-R_{01}{}^{2})e^{ink_{0}d}}{1-R_{01}{}^{2}e^{i2nk_{0}d}})$$

where *η* is the refractive index, the wave vector in free space is denoted as $k_{0}$, the prototype/slab thickness is denoted as *d*, and $R_{01}=\frac{z-1}{z+1}$. Solving (1) and (2) results in (3) as follows:

(3) $$z=\pm \sqrt{\frac{{\left(1+S_{11}\right)}^{2}-S_{21}{}^{2}}{(1-S_{11}{}^{2})-S_{21}{}^{2}}}$$

(4) $$e^{ink_{0}d}=X\pm i\sqrt{1-X^{2}}$$

where $X=\frac{1}{2S_{21}(1-S_{11}{}^{2}+S_{21}{}^{2})}$. As the material is deemed to be a passive medium, the impedance imaginary part should be greater than or equal to zero. Additionally, the real part of the refractive index (*η*) should be greater than or equal to zero. The *η* value of the material can be obtained from (5):

(5) $$n=\frac{1}{k_{0}d}[\{imaginary(\ln e^{ink_{0}d})+2m\pi \}-i\{real(\ln e^{ink_{0}d})\}]$$

where *m* is an integer value or the branch index of the real part of *η* in other studies [46]. It can be noted that in this extraction method, *m* = 0 was considered [36]. The values of *ε* and *μ* can be determined from the following expression [46,47]:

(6) $$\varepsilon =\frac{n}{z}$$

(7) μ=nz

**Figure 4:**
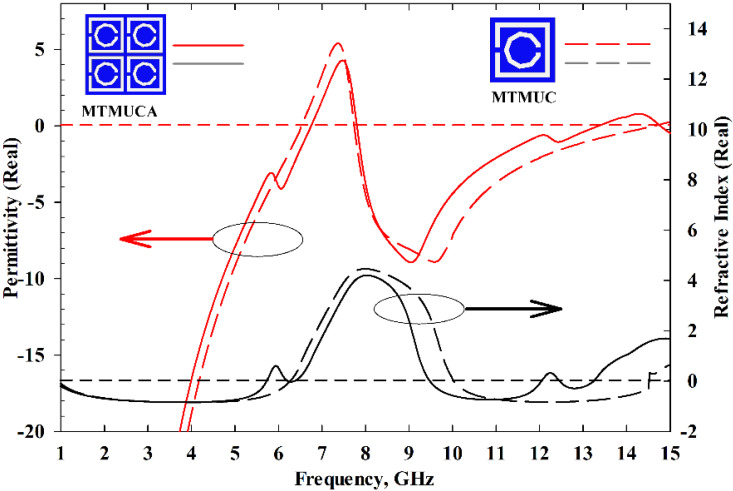
Permittivity and refractive index results of the MTMUC and MTMUCA structures.

Figure 4 illustrates the real part of the permittivity and refractive index. The MTMUC shows an ENG characteristic (*ε_r_* < 0) from 1 to 6.45 GHz, from 7.48 to 13.52 GHz, and from 14.96 to 15 GHz, whereas its NZRI characteristic (*η* < 0) is featured from 1 to 6.16 GHz, and from 9.35 to 13.44 GHz. On the other hand, the MTMUCA displays an ENG characteristic from 1 to 6.75 GHz, from 7.70 to 13.36 GHz, and from 14.73 to 15 GHz, whereas the NZRI characteristic is featured from 1 to 5.74 GHz, 6.21 to 6.37 GHz, 9.48 to 12.08 GHz, and 12.49 to 13.19 GHz. Based on the S-parameters and the MTM characteristics, the proposed MTMUC and MTMUCA can be used for stopband applications and microwave applications [28], e.g., C-band and Ku-band.

### 2.2. Metamaterial Working Principle

**Figure 5:**
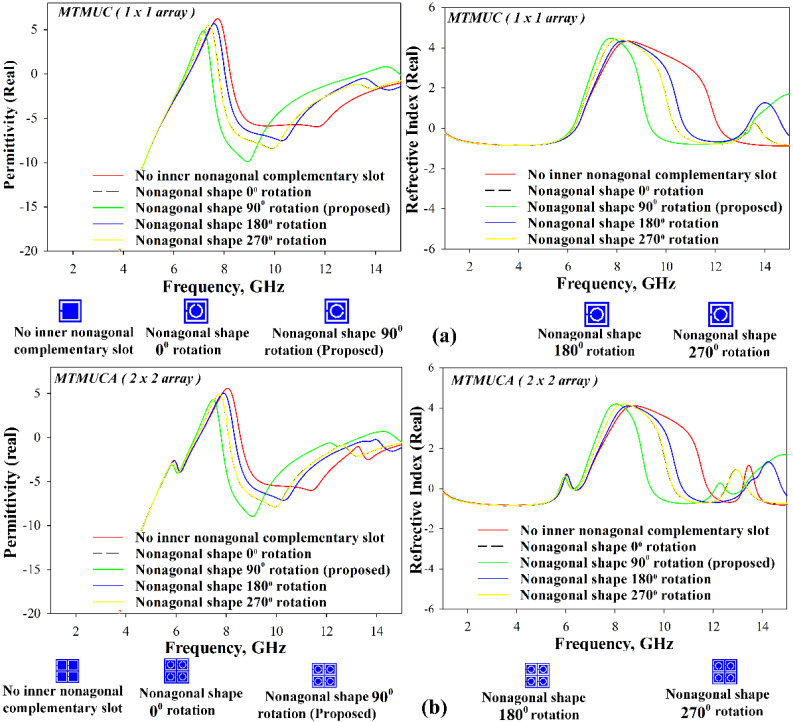
Analysis of relative permittivity and refractive index of (**a**) MTMUC and (**b**) MTMUCA.

To gain a further understanding of the MTM structure, a parametric study to analyze the effects of the nonagonal-shaped inner split conductor was performed, and its results are presented in Figure 5. In the absence of the inner conductor, a relatively narrower NZRI region can be observed for both the MTMUC and MTMUCA structures. Besides that, the structures’ inner nonagonal-shaped split ring was further evaluated when rotated at 0°, 90°, 180°, and 270° angles. The results are summarized in Table 3. When positioned at 0° and 270° rotation angles, the structure shows identical results for both MTMUC and MTMUCA. 

**Figure 6:**
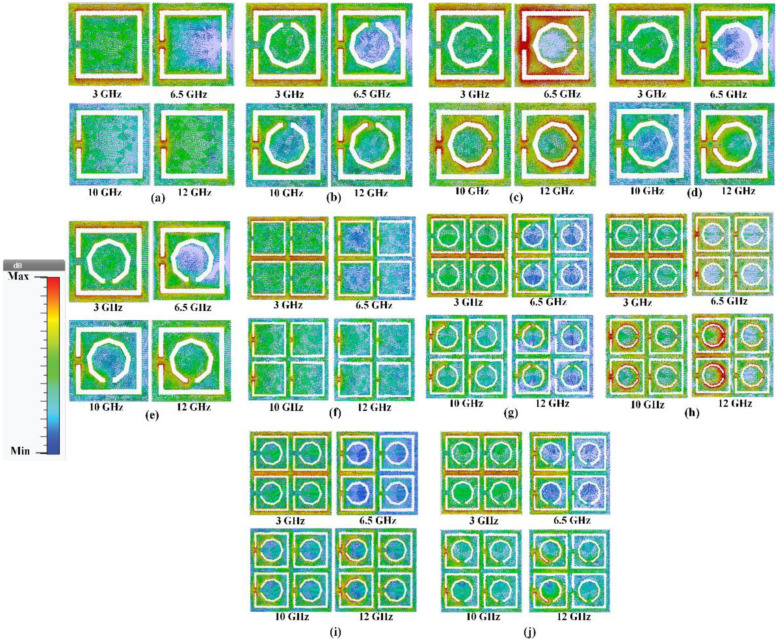
Surface current distribution for different MTM structures. MTMUC (1×1 array) (**a**) without the nonagonal-shaped inner split ring, and with (**b**) 0°, (**c**) 90°, (**d**) 180°, and (**e**) 270° rotation angles of the nonagonal-shaped inner split ring. MTMUCA (2 × 2 array) (**f**) without the nonagonal-shaped inner split ring, and with (**g**) 0°, (**h**) 90°, (**i**) 180°, and (**j**) 270° rotation angles of the nonagonal-shaped inner split ring.

To provide further insight into the working principles and properties of the proposed metamaterial, the simulated current distribution was analyzed and discussed. Figure 6 visualizes the surface current distribution at 3 GHz, 6.5 GHz, 10 GHz, and 12 GHz for the different types of MTM structures. The surface current density and direction are indicated by the colors and arrows, respectively. The concentration of the surface current at 3 GHz is almost indistinguishable, as shown in Figure 6a–e for MTMUC and Figure 6f–j for MTMUCA. On the contrary, when the nonagonal-shaped inner split conductor is absent in Figure 6a,f, a lower surface current density at 6.5 GHz, 10 GHz, and 12 GHz is observed compared to the structures with an inner conductor. Stronger surface currents are also observed at the edges of the rectangles and the nonagonal-shaped slot. For the MTMUC structures (in Figure 6a–e), the concentration of the surface current in Figure 6c shows the strongest surface current distribution. Likewise, among the MTMUCA structures (in Figure 6f–j), Figure 6h indicates the strongest surface current distribution. It is observed that in both cases, the position of the inner nonagonal slot shown in Figure 6c,h facilitated the achievement of stronger surface currents compared to the rest of the structures.

Comparison of the structures with different rotation angles indicated that the 90° rotated structure showed the strongest surface current distribution for both MTMUC and MTMUCA. This structure was chosen over the 0° or 270° rotated structure despite the latter being slightly better in terms of *ε* and NZRI BW. Therefore, the chosen structure was implemented in the UWB antenna to be studied further in the following section.

### 2.3. Antenna Design Geometry and Configurations

As it has previously been mentioned, the structure of the proposed antenna integrated with the MTMUCA is depicted in Figure 1. The planar monopole antenna was designed with a combination of rectangular and half elliptical-shaped patches, whereas two MTMUCAs were located 0.4 mm from both sides of the planar feedline. A partial ground plane was implemented on the reverse side of this feedline, and a 50 Ω SMA connector was connected at the end of the feedline. The overall dimension of the antenna was 33 × 30 × 3 mm^3^ (0.825λ_0_ × 0.75λ_0_ × 0.075λ_0_, where λ_0_ is the free space wavelength at 7.5 GHz, with the MTMUCA sized at 12.5 × 12.5 mm^2^ (0.312λ_0_ × 0.312λ_0_)). All dimensions are summarized in Table 2.

**Figure 7:**
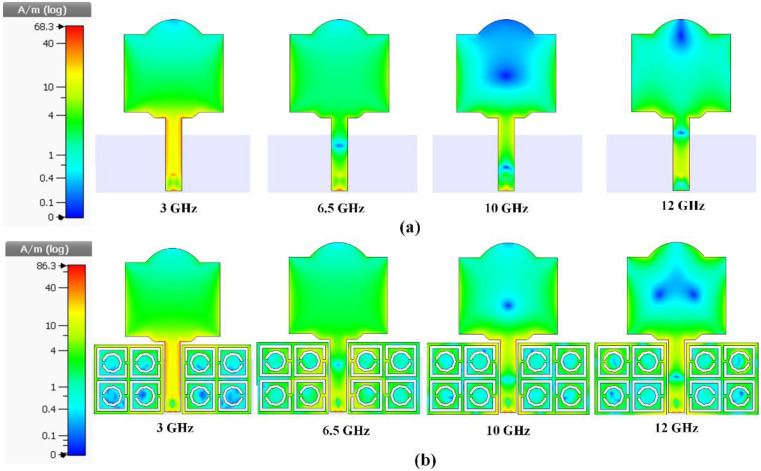
Surface current distribution. (**a**) Without the integration of the MTMUCA structure. (**b**) With the integration of the MTMUCA structure.

**Figure 8:**
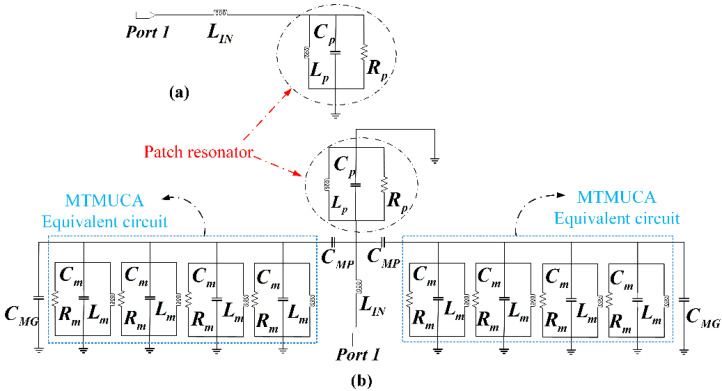
Equivalent circuit model. (**a**) Conventional planar monopole antenna with microstrip feedline antenna [48,49,50]. (**b**) Proposed antenna loaded with the MTMUCA demonstrated in Figure 1.

Figure 7 depicts the surface current distribution at 3 GHz, 6.5 GHz, 10 GHz, and 12 GHz with and without the MTMUCA structure integrated into the antenna. It is evident that the MTMUCA improved the current intensity. The circuit model of the structure was modeled based on [44,48,49,50], where the conventional planar monopole antenna model is illustrated in Figure 8a. The planar monopole antenna’s input impedance can be represented as an *RLC* circuit resonator near its resonance frequency, whereas the microstrip feedline can be expressed as a series inductor [44,48,50], resulting in the overall circuit model shown in Figure 8b. The CSRR-type MTMUC was modeled as a shunt *RLC* resonator tank (*R_m_*, *L_m_*, and *C_m_*) [44], which was designed to work at the frequencies of interest. The resistor *R_m_* indicates the dielectric and conductor losses, whereas the capacitance and inductance of the MTMCU are denoted as *L_m_* and *C_m_*, respectively. The short distance between the metallic ground plane and the MTMUCA was modeled as a capacitance, expressed as *C_MG_* [51], whereas *C_MP_* represents the capacitive coupling between the MTMUCA and microstrip feedline and/or patch resonator. 

The microstrip feed line behaves as an inductance, denoted as *L_IN_*. The CSRR acts as an electric dipole, and it mainly propagates along the *xy* plane within the substrate and radiates in the vicinity of the antenna.

To be more specific, the metamaterial’s EM energy could be coupled to the planar monopole antenna through *C_MP_*, whereas the radiation of the antenna was modeled using the radiation resistance *R_p_* [44]. The same applies to the coupling between the feedline and the MTMUCA. The coupling effect can be seen in Figure 7b, where a significantly increased current concentration is observed. Therefore, the implementation of the MTMUCA enables the operation of the antenna at the frequencies of interest and will be demonstrated in the next section.

## 3. Results and Discussion

**Figure 9:**
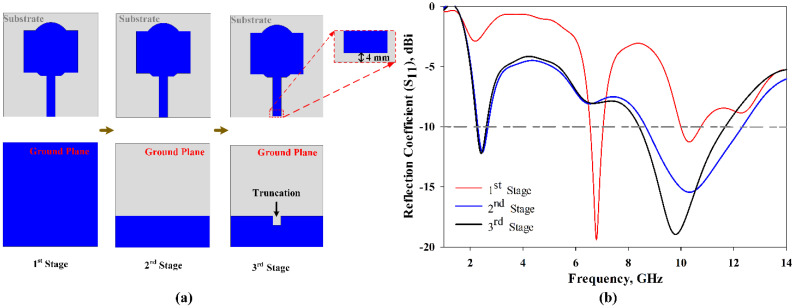
Evolution process of the conventional antenna in this study. (**a**) Evolution steps; (**b**) *S*_11_ results.

Figure 9 depicts the evolution process of the conventional antenna design in this study. In the first stage, as shown in Figure 9a, a full grounded patch with a combination of rectangular and half elliptical-shaped patches produces relatively narrow frequency bands, as shown in Figure 9b. In order to achieve a wider impedance bandwidth, the ground plane of the antenna was modified, as illustrated in the second stage of Figure 9b. From Figure 9b, it can be seen that the second stage has a wider impedance bandwidth compared to the first stage. Furthermore, in the third stage, a 0.4 mm gap was chosen to avoid a short circuit between the 50 Ω connector and the feedline of the planar monopole antenna. The truncation at the ground plane improved the impedance bandwidth slightly, where *S*_11_ resulted in being lower than −10 dB, showing over 2.33–2.6 GHz, and 8.52–12.3 GHz, which means FBW = 47% at the frequencies of interest. It can be noticed that the attained impedance bandwidth does not cover the complete UWB band allocated by the FCC. However, integration of the proposed MTMUCA with the conventional antenna can improve the overall performance. The related evidence can be found in the following results and discussion.

**Figure 10:**
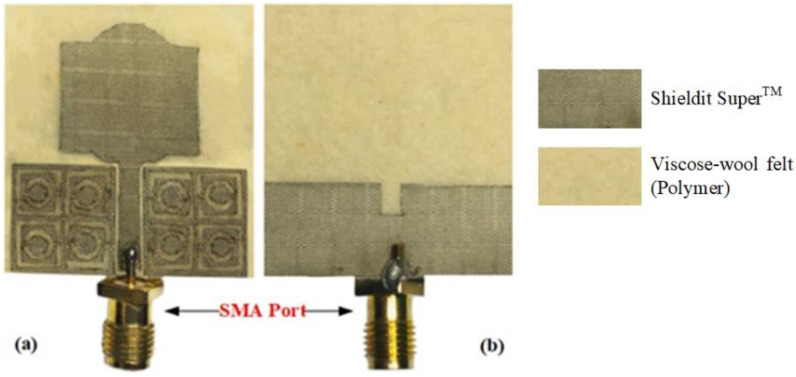
Prototype of the designed antenna: (**a**) front view, and (**b**) rear view.

**Figure 11:**
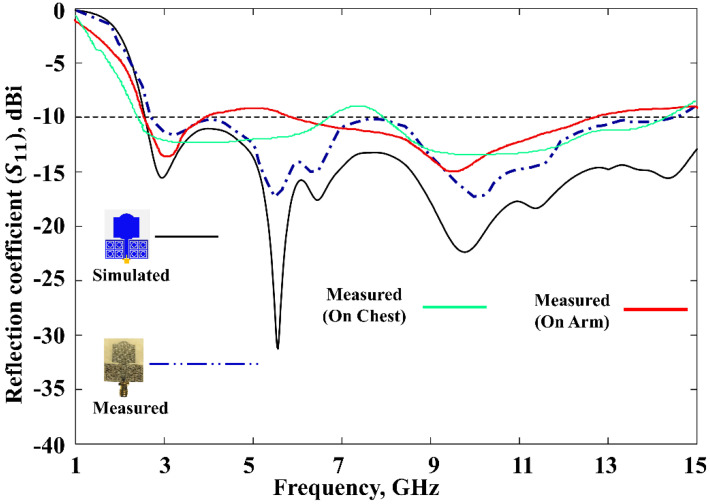
Reflection coefficients of the proposed antenna.

The fabricated prototype was used to validate this work, which is shown in Figure 10. The dielectric polymer material (viscose-wool felt) and conductive material (Shieldit Super^TM^) of the prototype were dimensioned by a laser cutting machine as a part of the fabrication process. A great deal of study has already been carried out on a variety of materials that have characteristics that render them appropriate for use as a substrate for conductive materials for antennas, conductive threads [52], conductive polymers [53], and conductive textiles [54]. However, in this study, viscose-wool felt was adopted since it provides easier fabrication with sufficient flexibility and enables strong adhesion with the conductive textile Shieldit Super^TM^. *S*_11_ measurement was performed using an Agilent E5071C Network Analyser (Agilent Technologies, Bayan Lepas, Penang, Malaysia) to ensure the simulated findings are accurate. The comparison of the proposed antenna between the simulated and measured *S*_11_ illustrated in Figure 11 indicates a good agreement. The simulation indicates a 10 dB impedance bandwidth from 2.55 to 15 GHz, which corresponds to an FBW of 142%. The measurement results indicate that this range is from 2.63 to 14.57 GHz, with an FBW of 138.84%. On the other hand, the antenna without the MTMUCA in Figure 9b (third iteration) shows a simulated FBW of 47%. Although the conventional antenna (considering the third iteration in Figure 9) does not work within the FCC region, by utilizing the unique characteristics of the metamaterial, the antenna performance could be enhanced. By utilizing the MTM on the conventional antenna, the antenna element’s radiation efficiency, gain, and overall performance can be improved.

**Figure 12:**
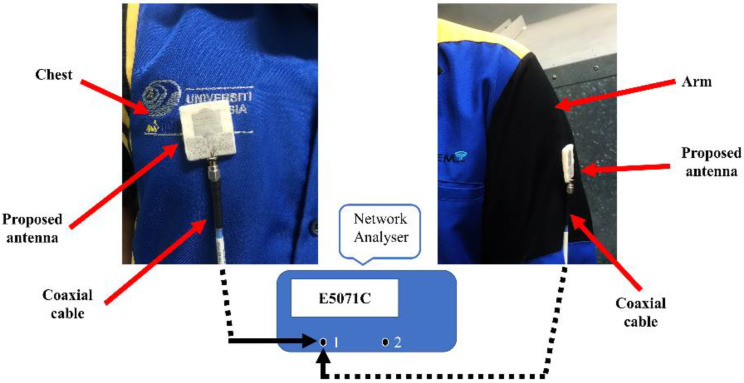
On-body measurement setup.

To rigorously analyze the performance of the proposed MTM-driven antenna prototype, body evaluation was further carried out with the help of a male volunteer (with a height of 1.72 m and weight of 84 kg), as shown in Figure 12. The prototype antenna was placed on two different places on the body (chest and arm). The related *S*_11_ results are presented in Figure 11, which indicate an excellent performance of the proposed antenna for on-body application. It can be noted that a 6 dB reflection coefficient is frequently used in the manufacturer’s specification, as reported in [55,56]; hence, the obtained performance is sufficient for practical application.

**Figure 13:**
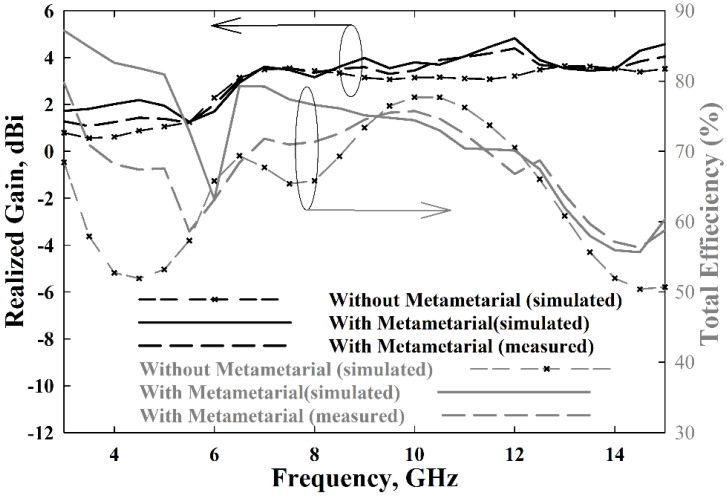
The proposed antenna’s realized gain and radiation efficiency.

Figure 13 shows the antenna gain and total efficiency over the frequency. In simulations, an average realized gain of 3.3 dBi was achieved with the MTMUCA, whereas it was 2.7 dBi without the MTMUCA. A maximum gain of 4.83 dBi and 3.64 dBi could be obtained with the proposed antenna with and without the MTMUCA, respectively. On the other hand, the average measured gain of the proposed antenna is 3.04 dBi, and the maximum peak gain is 4.4 dBi. The maximum total efficiency achieved is 87% and 79.5% for the antenna with and without MTMUCA implementation, respectively, in simulations. The attainable average total efficiency of the proposed antenna is approximately 73%, whereas the antenna without the MTM indicated levels of around 69%. The measurements show a maximum total efficiency of 80%, and the average efficiency is 68.2%, for the proposed integrated antenna.

**Figure 14:**
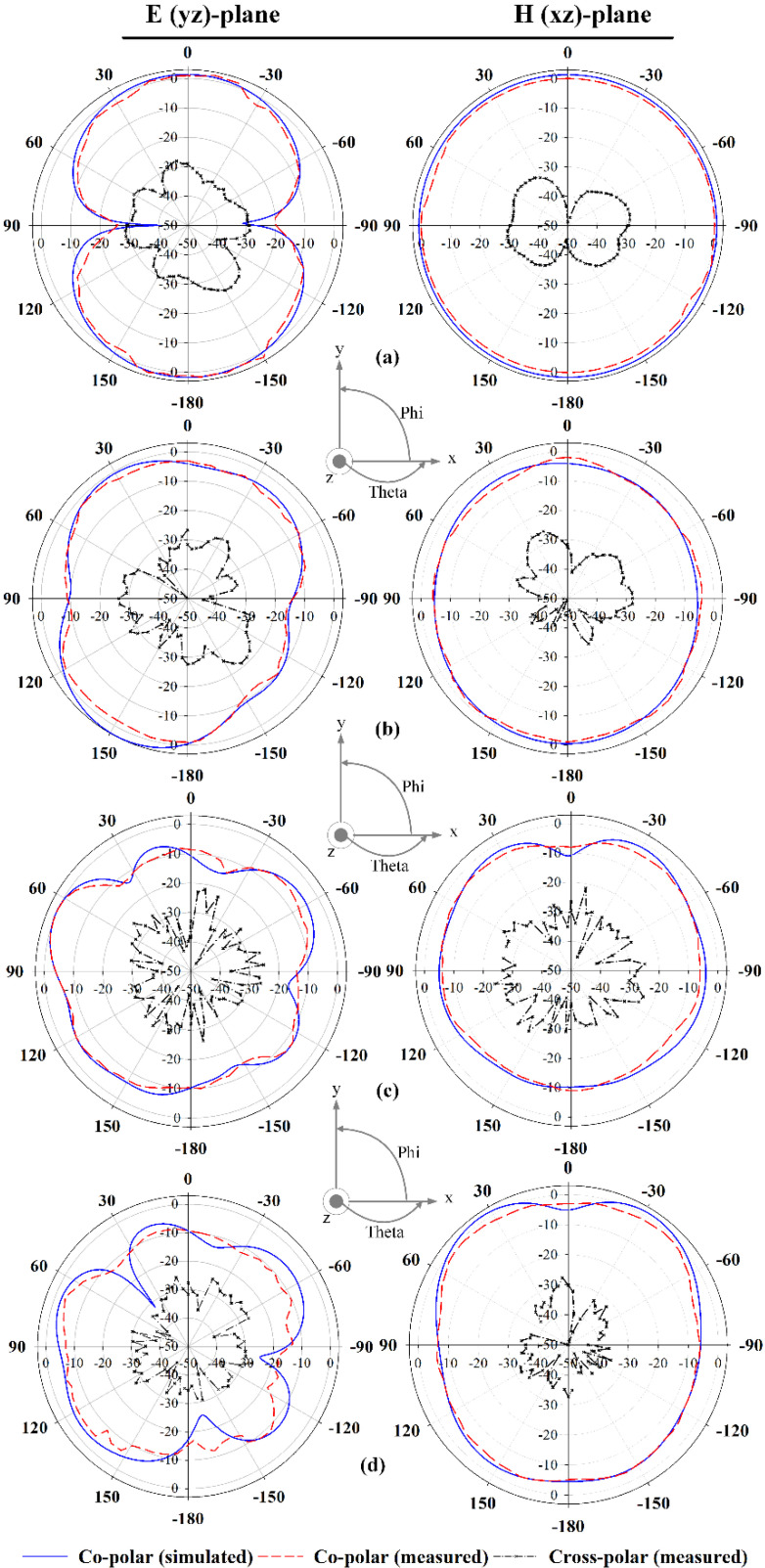
Radiation patterns of the proposed antenna at: (**a**) 3 GHz, (**b**) 6.5 GHz, (**c**) 10 GHz, and (**d**) 12 GHz.

Figure 14 illustrates the radiation characteristics of the proposed antenna. The simulated and measured radiation patterns of the E-plane (*yz*-plane) and H-plane (*xz*-plane) were performed at four different frequencies, i.e., 3 GHz, 6.5 GHz, 10 GHz, and 12 GHz, indicating very good agreement. An omnidirectional radiation pattern can be observed at 3 GHz. Meanwhile, at 6.5 GHz, 10 GHz, and 12 GHz, an omnidirectional pattern can be seen in the H-plane, whereas the E-plane shows a bidirectional radiation pattern. Slight discrepancies between the simulations and measurements can be observed due to fabrication inaccuracies.

## 4. Conclusions

A compact textile UWB planar monopole antenna integrated with an MTMUCA featuring ENG and NZRI properties was proposed and studied. The MTM-integrated antenna prototype was fabricated using flexible polymer viscose-wool felt as the substrate (since it provides easier fabrication with sufficient flexibility) and Shieldit Super^TM^ as the conductive element. The proposed MTMUC structure consists of a unique combination of square- and nonagonal-shaped CSRR-type MTMs. The proposed MTMUCA and MTMUC exhibited SNG properties in different frequency bands. The MTMUCA structure featured an 11.68 GHz ENG bandwidth and an 8.5 GHz NZRI bandwidth. This work also presented an equivalent circuit design to illustrate the principles of the overall structure. The implementation of the MTMUCA into the antenna design was proven experimentally to improve the antenna gain, efficiency, and bandwidth. Measurements showed an FBW of up to 138.84%, from 2.63 to 14.57 GHz, with a peak gain and total efficiency of 4.4 dBi and 80%, respectively. The proposed design can potentially be applied for wearable applications, where future research could be carried out to investigate the feasibility of on-body application, specifically for breast cancer detection.

## Figures and Tables

**Table 1:** Comparison of the proposed design with relevant previous work in the literature.

Reference	Size (mm^3^) λ_0_ = 40 mm	Operating Frequency Range (GHz)	Metamaterial Structure/Technique	Fractional Bandwidth (FBW) (%)	Antenna Peak Gain (dBi)	Remarks
[37]	52.5 × 52.5 × 20 (1.313 λ_0_ × 1.313 λ_0_ × 0.5 λ_0_)	2.5–13.8 BW = 11.3	Flexible AMC metamaterial	138.65	9	Huge gap between the antenna and separate MTM layer made the overall antenna size bigger, complicated, and practically unusable.
[38]	43 × 40 × 2 (1.075 λ_0_ × 1 λ_0_ × 0.05 λ_0_)	1.8–10 BW=8.2	Semicircular ring resonator in the patch	139	5.09	Comparatively low BW and large antenna size.
[39]	20 × 12 × 0.8 (0.5 λ_0_ × 0.3 λ_0_ × 0.02 λ_0_)	2.4–10 BW = 7.6	Metamaterial	122.58	3.456	Antenna is compact in terms of size, but the BW and peak gain are not superior.
[17]	105 × 91 × 7.9 (2.625 λ_0_ × 2.275 λ_0_ × 0.1975 λ_0_)	3.5–12.40 BW=8.9	Metasurface	114	9.1	Antenna size is large when including MTM. Increased design complexity with the use of multiple layered substrates.
[40]	50 × 43 × 14.95 (1.25 λ_0_ × 1.075 λ_0_ × 0.374 λ_0_)	2.3–16 BW = 13.7	MNG metamaterial	149.73	8	The antenna has separate MNG MTM layer. Multilayer antenna design and huge gap between the antenna and MTM layer made the design complicated.
[34]	48 × 36 × 6 (1.2 λ_0_ × 0.9 λ_0_ × 0.15 λ_0_)	8.2–13 BW=4.8	AMC metamaterial	45	7.04	Low BW and does not cover required FCC BW. Large antenna size incorporated on a separate AMC layer.
Proposed work	33 × 30 × 3 (0.825 λ_0_ × 0.75 λ_0_ × 0.075 λ_0_)	2.55–15 BW= 12.45	SNG/NZRI metamaterial	142	4.84	Compact size and wide operational bandwidth and FBW.

**Table 2:** Parameter dimensions of the proposed antenna.

Para.	Value (mm)	Para.	Value (mm)	Para.	Value (mm)
Ls	33.00	c	3.68	x	6.50
Ws	30.00	d	2.60	y	5.50
Wp	17.20	e	4.20	r	1.75
Lp	13.40	f	1.81	g_2_	3.00
Wg	13.70	g	1.56	g_3_	2.60
Lg	10.00	g_1_	0.5	Wf	2.80

**Table 3:** Negative permittivity and negative refractive index results of different types of MTM structures.

MTM Structure			Negative Permittivity Band (GHz)	Negative Refractive Index Band (GHz)
Array Structure		Inner Nonagonal-Shaped Split Ring Rotation Angle (Degrees)		
**1 × 1 (MTMUC)**	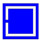 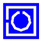 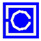 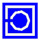 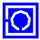	–	1–6.58 8.2–15	1–6.33 12.17–15
		0	1–6.50 7.78–15	1–6.21 10.39–13.33 13.78–15
		90	1–6.45 7.48–13.52 14.96–15	1–6.16 9.35–13.44
		180	1–6.57 8.02–15	1–6.31 10.79–13.26 14.80–15.00
		270	1–6.50 7.78–15	1–6.21 10.39–13.33 13.78–15
**2 × 2 (MTMUCA)**	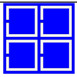 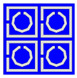 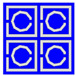 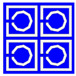 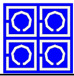	–	1–6.88 8.57–15	1–5.79 6.28–6.56 11.79–13.19 13.74–15
		0	1–6.80 8.11–15	1–5.76 6.23–6.43 10.39–12.5 13.39–15
		90	1–6.75 7.70–13.36 14.73–15	1–5.74 6.21–6.37 9.48–12.08 12.49–13.19
		180	1–6.88 8.33–15	1–5.81 5.56–6.29 10.8–13.28 14.83–15
		270	1–6.8 8.11–15	1–5.76 6.23–6.43 10.39–12.5 13.39–15

## Data Availability

This study did not report any data.
